# Do synthetic absorbable sutures cause less uterine tissue reactions than catgut in bovine caesarean sections?

**DOI:** 10.18849/ve.v11i4.742

**Published:** 2026-11-09

**Authors:** Tom Woods

**Affiliations:** University of Edinburgh United Kingdom

**Keywords:** ABDOMINAL, BOVINE, CAESAREAN, CALVING, CATGUT, SUTURE, UTERINE

## Abstract

**PICO question:**

In bovines undergoing caesarean section does the use of synthetic absorbable uterine sutures compared with catgut result in less uterine tissue reactions?

**Clinical bottom line:**

**Category of research:**

Treatment.

**Number and type of study designs reviewed:**

Three studies were critically appraised. Two were non-randomised controlled trials and one was a randomised controlled trial.

**Strength of evidence:**

Weak.

**Outcomes reported:**

One study found no difference in the incidence of uterine adhesions between plain catgut and polyglactin 910. Two other studies, which were both conducted on the same group of animals using chromic catgut and polyglycolic acid (PGA), demonstrated a lower incidence and severity of uterine adhesions, as well as reduced histopathological indicators of uterine tissue damage, with polyglycolic acid sutures compared to chromic catgut.

**Conclusion:**

There is limited evidence concerning the difference in uterine tissue reactions caused when using catgut or a synthetic uterine suture material in bovine caesarean surgeries. The lack of comparability between the relevant studies in terms of population characteristics and type of suture material used (chromic and plain catgut) does not allow a clear conclusion to be made. In addition, uterine adhesion formation is likely to be multifactorial and there is no overall clear indication that the use of synthetic absorbable suture materials is superior in terms of reduced uterine tissue reactions. There are several other papers of interest which may provide further evidence, but these are not available in English and are therefore outside the scope of this Knowledge Summary.

How to apply this evidence in practice

The application of evidence into practice should take into account multiple factors, not limited to: individual clinical expertise, patient’s circumstances and owners’ values, country, location or clinic where you work, the individual case in front of you, the availability of therapies and resources.

Knowledge Summaries are a resource to help reinforce or inform decision making. They do not override the responsibility or judgement of the practitioner to do what is best for the animal in their care.

## Clinical scenario

During a clinical audit of bovine caesareans in your practice, it becomes apparent that there are some differences amongst your colleagues in their approach to the procedure. One of these is the choice of suture material. Some of your colleagues use chromic catgut and are not convinced of the benefits of switching to a more expensive synthetic suture material. As a recent graduate, you prefer to use Vicryl (polyglactin 910), as you remember being taught that synthetic absorbable sutures cause fewer tissue reactions in small animal surgeries than catgut. You wonder if this is also the case in bovine caesareans, so you decide to find out if there is any evidence to support your claim and persuade your colleagues to change to using synthetic absorbable sutures.

## The evidence

The strength of the evidence is weak. The three studies (Dhindsa et al., 2010a; Dhindsa et al., 2010b; Mijten et al., 1997) are controlled trials (one randomised and two non-randomised). However, two of these studies (Dhindsa et al., 2010a; Dhindsa et al., 2010b) were carried out on the same group of animals and assessing different outcomes. Four different suture materials were used across the three studies, with three head-to-head comparisons made. There were limitations to the study designs, some of which were related to the practical aspects of carrying out bovine caesarean surgery. Additionally, the inclusion of different species of bovines (buffalo and *bos taurus*) as well as the variation in suture material used (catgut and chromic catgut), makes direct comparisons between the outcomes difficult.

## Summary of the evidence

**Dhindsa et al. (2010a):** Alterations in uterine and peritoneal fluid cytology as well as uterine histopathology following caesarean operation with reference to suture material and intra-peritoneal lubricant in bovines **Aim: **To determine the differences in effect between two suture materials (polyglycolic acid and chromic catgut) and the use of an intra-abdominal lubricant (sodium carboxy methyl cellulose) on uterine and peritoneal fluid, and uterine histology associated with inflammatory responses following caesarean section in bovines.

Population:	Cows and buffaloes undergoing caesarean section at Guru Angad Dev Veterinary and Animal Sciences University, Ludhiana, Punjab, India.
Sample size:	41 (10 cows and 31 buffaloes).
Intervention details:	The animals were divided into four groups based on whether chromic catgut (CC) or polyglycolic acid (PGA) was used for uterine sutures both with and without sodium carboxyl methyl cellulose (SCMC) used as an intra-abdominal lubricant: CC (n = 9), CC+SCMC (n = 10), PGA (n = 10), PGA+SCMC (n = 12). The surgeries were performed in right-lateral recumbency and the uterus was sutured using a Cushing pattern. 5 litres of saline was given intravenously to all animals at the time of surgery and repeated daily until day 6 postsurgery. All animals were treated with the same twice-daily antimicrobial therapy of gentamicin 5 mg/kg and streptomycin 2.5 g (intramuscularly), repeated on subsequent days for an unspecified duration. Additional medications including adrenochrome monosemicarbazone, dexamethasone, meloxicam, multivitamins, pheniramine maleate and ruminotorics were administered. However, the specific dose, route, and frequency is not stated for each, and it is unclear if all animals received the same treatments.
Study design:	Non-randomised controlled trial.
Outcome Studied:	Histopathological changes on uterine tissue biopsies collected at the time of surgery from the incision site and then on day 6 with a biopsy punch from the gravid horn. Histology was assessed for epithelial/sub-epithelial and endo-/myometrial damage on a three-point scale, ranging from mild to severe. The superficial inflammatory changes were also characterised as either acute or chronic. Uterine and peritoneal fluid samples were collected at the time of surgery and at 24 hours later by uterine catheter and abdominocentesis respectively. The findings of these were not relevant to the PICO question and are therefore not discussed further.
Main Findings (relevant to PICO question):	All histopathological changes in the CC only group increased in severity before and after surgery. There were minimal histopathological changes in the CC+SCMC and PGA groups pre- and post-surgery. However, the severity of epithelial congestion and haemorrhage in the PGA+SCMC group reduced and inflammatory changes remained acute.
Limitations:	Postsurgery tissue biopsies were collected from anywhere in the gravid horn rather than specifically at the incision site, so it is difficult to know if the findings are directly related to the suture material used or due to trauma occurring during parturition and surgical handling of the uterus. The additional assessment of using the SCMC lubricant added another variable which also made the individual group sizes small. The study included a mix of buffaloes (*Bubalus bubalis*) and cows (likely *Bos taurus *or *Bos indicus* but not stated) with the results not separated for each. It is unclear if the surgeries were performed by the same surgeon or by multiple surgeons. It is not stated whether any of the surgeries were affected by significant complications or obvious contamination of the abdominal cavity. It is not stated if cows were spread evenly across the four groups based on time since onset of parturition. The influence of this could have affected the results in terms of histopathological changes. The criteria used to characterise the histopathological changes as mild, moderate and severe is not described. It is unclear if the person assessing the histology specimens was blinded as to which group the samples came from. The results indicate that the CC only group had more oedema and haemorrhage presurgery, but the reasons for this are not included in the discussion. Statistical analysis was not carried out on the histopathological data (although this can be difficult with ordinal data). There are inconsistencies in the description of postsurgical drug administration. It is suggested that all animals received the same medications, but this is not explicitly stated and the duration of treatment, dose, and route is not made clear in every case.

**Dhindsa et al. (2010b):** Prevention of uterine adhesion formation following caesarean section in bovines **Aim: **To determine the differences in effect between two suture materials (polyglycolic acid and chromic catgut) and the use of an intra-abdominal lubricant (sodium carboxy methyl cellulose) on the incidence of uterine adhesion formation following caesarean section in bovines.

Population:	Cows and buffaloes undergoing caesarean section at Guru Angad Dev Veterinary and Animal Sciences University, Ludhiana, Punjab, India.
Sample size:	41 (10 cows and 31 buffaloes).
Intervention details:	Bovines were divided into four groups based on whether chromic catgut (CC) or polyglycolic acid (PGA) was used for uterine sutures both with and without sodium carboxyl methyl cellulose (SCMC) used an intra-abdominal lubricant: CC (n = 9), CC+SCMC (n = 10), PGA (n = 10), PGA+SCMC (n = 12). Cows were also grouped based on the duration of dystocia:< 12 h (n = 12), 12–36 h (n = 11) and > 36 h (n = 18). It was attempted to distribute animals in each of these three sub-groups equally amongst the four main groups. The surgeries were performed in right-lateral recumbency and the uterus was sutured using a Cushing pattern in each case. 5 litres of saline were given intravenously to all animals at the time of surgery and repeated daily until day 6 postsurgery. All animals were treated with the same twice-daily antimicrobial therapy of gentamicin 5 mg/kg and streptomycin 2.5 g (intramuscularly), repeated on subsequent days for an unspecified duration. Additional medications including adrenochrome monosemicarbazone, dexamethasone, meloxicam, multivitamins, pheniramine maleate and ruminotorics were administered. However, the specific dose, route and frequency is not stated for each, and it is unclear if all animals received the same treamtents.
Study design:	Non-randomised controlled trial.
Outcome Studied:	Objective assessment using defined parameters of the presence and severity of periuterine adhesions by manual rectal palpation at between 30 to 60 days postsurgery. The visual and manual assessment of uterine adhesion presence and severity at postmortem examination in animals which died at the hospital after surgery.
Main Findings (relevant to PICO question):	In the CC only group, all 7 of the animals developed severe uterine adhesions after surgery compared to only 6/10 (60%) in the PGA only group. Animals from the original group were excluded because they were sold by the farmer approximately 20–30 days post-caesarean. In the group where PGA was used without SCMC, there was a statistically significant reduction in the number of animals that developed any adhesions (P = < 0.05) when compared with the CC only and CC+SCMC groups. The group where PGA and SCMC were used had a higher percentage of cows without or with only mild adhesions (P = < 0.05) and a lower percentage of cows with severe adhesions than either the CC or the CC+SCMC groups (P = < 0.05). 13/15 (86.7%) of cows developed severe adhesions where the dystocia had lasted more than 36 hours compared to only 3/10 (30%) in those lasting under 12 hours (P = < 0.05).
Limitations:	It is unclear if the surgeries were performed by the same surgeon or by multiple surgeons. The study included a mix of buffaloes (*Bubalus bubalis*) and cows (likely *Bos taurus* or *Bos indicus* but not indicated) with the results not separated for each. The overall sample size was small, leading to small numbers around 10 animals in each of the four groups. The animals were assigned to the four groups partly based on the duration of parturition, but it is not clearly stated what other factors were considered. Therefore, assumed to be non-randomised. Animals were assessed at different time points postsurgery and the information of this distribution is not given. It is not stated how many animals died and were assessed at postmortem and at how many days postsurgery they died. These animals may not have had time for adhesions to develop if they died shortly after surgery. It is not stated if the outcome assessor is blinded as to which groups the cows belonged to. Five animals were excluded during the trial as they were sold before assessment was carried out, reducing the group sizes even further. The additional assessment of using the SCMC lubricant added another variable which itself was demonstrated to affect adhesion formation and also made the individual group sizes smaller. It is not stated whether any of the surgeries were affected by significant complications or obvious contamination of the abdominal cavity. Only periuterine adhesions were assessed and not intrauterine adhesions. There are inconsistencies in the description of postsurgical drug administration. It is suggested that all animals received the same medications, but this is not explicitly stated and the duration of treatment, dose, and route is not made clear in every case.

**Mijten et al. (1997):** Comparison of catgut and polyglactin 910 for uterine sutures during bovine caesarean sections **Aim: **To determine the effect of two suture materials (polyglactin 910 and plain catgut) on the incidence of uterine adhesion formation following caesarean section in bovines.

Population:	Dairy and beef cattle undergoing caesarean section in practice conditions.
Sample size:	153 cows.
Intervention details:	Cows were randomly allocated into two groups where either plain catgut or polyglactin 910 (Vicryl) was used for the uterine sutures (78 and 75 in each respectively). A variety of dairy and double-muscled beef breeds were used as well as primiparous and multiparous cows. Five surgeons participated. Two of these closed the uterus with the Utrecht pattern and three closed with the Lembert method. The animals assigned to each suture material were spread across the two closure pattern groups. All cows received 10,000 IU penicillin G, 10 mg dihydrostreptomycin (intramuscularly) and an intrauterine antibiotic bolus containing 1 g chlortetracycline. During surgery, some fractious cows received 10–15 mg xylazine (intravenously) and some received 100 mg isoxsuprine (IM) to aid uterine manipulation. Postsurgery, some cows also received 50 IU oxytocin (IM), with the distribution fairly even between the two suture material groups. All surgeries were performed on standing animals undergoing caesarean section for the first time and cows with obvious contamination occurring during surgery were excluded.
Study design:	Randomised controlled trial.
Outcome Studied:	Objective assessment of the presence of periuterine adhesions by manual rectal palpation at 5 weeks postsurgery. The uterus was retracted and each horn turned over. If this was not possible, adhesions were recorded if palpated along the incision site. The assessor was blinded as to which group the cows belonged to.
Main Findings (relevant to PICO question):	48/78 (61%) of cows developed uterine adhesions in the catgut group and 47/75 (63%) developed adhesions in the Vicryl group. Overall, 95/153 (62%) of cows in the study developed uterine adhesions across both groups. There was no statistically significant correlation between the presence of uterine adhesions and the type of suture material used.
Limitations:	Two different suture patterns were used in the study which adds an extra variable. Five different surgeons performed the surgery which adds an additional variable and appears to have had a significant influence on the incidence of adhesions. It is not made clear if all the surgeries were carried out at one location or at multiple practices, which may have had different handling facilities. Cows were not evenly distributed amongst the surgeons using different suture patterns. The duration of parturition or the surgery were not recorded, which could have influenced the results. The method of randomisation in assigning the cows to the suture material groups is not described. Only periuterine adhesions were assessed (not intrauterine) and the adhesions were not graded by severity. The duration of parturition before surgery is not stated or assessed, which could have impacted the outcome in each case. There was a statistically significant difference (P = < 0.05) in the incidence of uterine adhesions between two of the surgeons using the Utrecht method for both suture materials.

## Appraisal, application and reflection

From the available evidence relating to the PICO question (Dhindsa et al., 2010a; Dhindsa et al., 2010b; Mijten et al., 1997), it does not appear that there is a clear difference between the use of catgut and synthetic absorbable suture materials in the bovine caesarean surgery when looking at the degree of uterine tissue reactions caused. The study by Mitjen et al. (1997) found no difference between plain catgut and polyglactin 910, whereas the studies by Dhindsa et al. (2010a; 2010b) found an increased severity and incidence of periuterine adhesions, as well as a higher severity of uterine histopathological changes with chromic catgut compared to polyglycolic acid. Studies in other species such as rats (Bakkum et al., 1995; Biondo-Simões et al., 2018) show similar mixed conclusions when looking at differences in peritoneal adhesion formation between plain catgut and synthetic sutures. One study by Bakkum et al. (1995) found a higher incidence and severity of uterine adhesion formation with plain catgut compared with polyglactin 910, whereas another study by Biondo-Simões et al. (2018) found the opposite, with a higher severity and incidence with polyglycolic acid. There are several other studies on bovine caesareans which were likely to be relevant to the PICO question and with large sample sizes, but are unfortunately not available in English (Zaag, 1995; Bohy, 2001; Boucoumont et al., 1979). A review of these studies in addition to those included in this Knowledge Summary may provide a stronger body of evidence and a clearer answer to the PICO question.

The clinical significance of uterine adhesions is that they may cause pain, intestinal obstructions, and affect the prognosis of the cow returning to the breeding herd (Newman & Anderson, 2005). A prospective study of 103 cows undergoing caesarean sections showed that although the survival rate was favourable (80%), only 55% calved again in subsequent years (Lyons et al. 2013). Although it is useful to look at uterine adhesion formation and histopathological tissue changes, the main clinical outcomes of interest are firstly survival and then the ability to return to normal fertility. It would be beneficial to carry out more studies in this area to assess any potential benefits of synthetic suture materials, particularly with regards to the effect on fertility after surgery. In the Knowledge Summary author’s experience, the accuracy of the detection of uterine adhesions on rectal examination could also be questionable. In addition, only periuterine adhesions are being assessed in these studies. Intrauterine adhesions could also be present, which are also likely to impact the future fertility of the animal. It would be more comprehensive to perform a postmortem examination to fully and accurately assess adhesion formation, although the practicality of doing this would be difficult.

The studies by Dhindsa et al. (2010a; 2010b) included a reasonable sample size for a veterinary study of 41 animals. However, the studies also chose to measure the outcome of an additional variable by assessing the effect of adding an intra-abdominal lubricant as well as the different suture material. It was demonstrated that the lubricant also had an effect on periuterine adhesion formation, which makes it more difficult to assess the effect of the two suture materials. This also meant that the population was split into four groups with very small numbers of animals in each. This impacts the power of the study and means that a more cautious interpretation of the results must be taken. It is also notable that 31 of the 41 animals in these studies were buffaloes (*Bubalus bubalis*), rather than the *Bos taurus* cattle most commonly encountered in the UK. The specific breeds of cattle were not stated in any of the studies. A distinction is made by Mitjen et al. (1997) between ‘double-muscled’ and ‘dairy’ cattle, but there is no analysis made of the difference in uterine adhesion formation between the two groups. Dhindsa et al. (2010a; 2010b) make no reference to breed and also do not offer any insight into any different outcomes between buffalo and cows. However, the small sample sizes of the groups in these two studies would likely preclude any statistically significant findings being made. There is no available evidence to suggest differences in adhesion formation between different bovine species or breeds and there would be no significant differences in surgical approach, but the influence of these variables on the results and the clinical relevance cannot be ruled out.

Other variables in these studies such as parturition duration (Dhindra et al., 2010b) and the surgeon performing the surgery (Mitjen et al., 1997) also had a significant effect on the formation of uterine adhesions postsurgery. It was not made clear in Dhindra et al. (2010b) how many surgeons were involved and if there was any difference between them, whereas Mitjen et al. (1997) did not record the duration of parturition for each surgery. It is therefore difficult to be confident that the results have not been influenced by these variations in the sample populations. Many factors can increase the risk of uterine adhesions during surgery including uterine tears, wound dehiscence, presence of blood clots and contamination of the serosal surface (Kumar et al., 2022). Whilst the quality of the studies could have been improved by attempting to control as many of these variables as possible and minimise their influence, the unpredictable nature of the bovine caesarean surgery makes the practicality of this challenging. When appraising the results of all studies, it is important to be aware that the adhesion formation is likely to have been multifactorial and suture material choice may not have been the most important factor in each case.

The study by Dhindsa et al. (2010a) found that catgut without an intraperitoneal lubricant induced a higher severity of histopathological changes than polyglycolic acid in samples taken pre- and postsurgery. The bovine caesarean is inherently a traumatic surgery which requires robust uterine tissue handling and can have varying levels of uterine tissue trauma depending on the cause of dystocia. However, none of the three studies recorded the cause of dystocia for each case. Recording these details and excluding animals that experienced severe uterine trauma could have been an improvement to the study designs. As catgut is a natural biological material, absorbed through inflammatory processes rather than hydrolysis as with synthetic materials (Gomes & Reis, 2004), it may not be surprising that more severe inflammatory changes would be seen in the catgut group. However, a study comparing suture materials in rats by Biondo-Simões et al. (2018) found no correlation between histopathological inflammatory changes and peri-uterine adhesion formation. The clinical significance of the difference in histopathological changes between the two suture materials in terms of adhesion formation is therefore questionable.

The types of suture material used in all three studies differed, which may have had an effect on the outcomes. Mitjen et al. (1997) used plain catgut and polyglactin 910, whereas Dhindsa et al. (2010a; 2010b) used chromic catgut and polyglycolic acid. Polyglycolic acid and polyglactin 910 both have similar properties and absorption rates. However, plain catgut has a rapid absorption profile, whereas chromic catgut is treated with chromium salts to slow the degradation rate (Singh & Maxwell, 2006). The fact that the plain catgut would have been absorbed faster could have meant that there was less time for suture-related uterine adhesions to develop and therefore less difference seen against the synthetic material than in the study using chromic catgut. The use of plain catgut has largely been replaced by chromic catgut, so the findings of the study by Mitjen et al. (1997) may not be directly translatable in a modern clinical setting. Suture size was also not described in any of the studies, which may have had an effect on absorption time.

The evidence available does not yet make a compelling argument for practitioners to move away from catgut for bovine caesareans. Importantly, there are practical considerations when choosing between natural or synthetic suture materials. Synthetic sutures are difficult to source in sizes above 2 metric, whereas a significantly thicker 7 metric chromic catgut is commonly preferred for bovine caesareans. Although synthetic sutures often have a more reliable tensile strength than catgut (Singh & Maxwell, 2006), the thinner material can be more likely to tear through friable uterine tissue during suturing. This effect can also be exaggerated if a braided material is used, such as polyglycolic acid (PGA) or Vicryl (Mitjen et al. 1997). Increased cost is also frequently mentioned in the literature as a prohibiting factor in the use of synthetic materials. However, in reality this would add a proportionally small cost onto the surgery when compared to using catgut. If improved animal welfare and production outcomes were clearly demonstrated to be better with synthetic sutures, cost would no longer be an appropriate reason to avoid them.

Depending on the country, legal requirements may be the major deciding factor when choosing between catgut and synthetic materials. After the Bovine Spongiform Encephalopathy (BSE) crisis of the 1990s, the use of catgut was banned in the European Union and also in Japan and its use mostly replaced with synthetic sutures (Langley-Hobbs, 2014).

Further evidence to answer this clinical question could be gathered informally in-practice through clinical auditing. This could either be done prospectively or retrospectively by looking back through practice records. Technique variations and suture materials used by different surgeons can be easily recorded and combined with the follow up data. This information can then be used to inform best practice and to create practice guidelines on the procedure. Outcomes such as the survival rate, culling rate, and the success rate of returning to pregnancy are likely to be the most clinically useful and easily measurable. However, the follow-up of these cases can be challenging as animals are sometimes sold or the outcomes not recorded by the farmer. Measuring outcomes such as the production of a live calf in the year following a caesarean relies on careful recording and the willingness to follow up cases at later points in time. Effective collaboration between colleagues and clients is required to achieve this.

## Methodology

**Search Strategy:** 

Databases searched and dates covered:	CAB Abstracts on OVID platform: 1910 to Week 14 2025PubMed on NCBI platform: 1910 to April 2025Web of science on Clarivate: 1900 to April 2025
Search strategy:	CAB Abstracts: (cow? OR bovine? OR cattle? OR bos) (catgut OR polyglactin OR polyglycol* OR vicryl OR suture material? OR polydioxanone OR poliglecaprone OR PDS OR monocryl OR PGA) (c?esarean OR laparotom*) 1 AND 2 AND 3 PubMed: (bovine OR bovines OR cow OR cows OR cattle OR bos) (catgut OR polyglactin OR vicryl OR polyglycol* OR suture material OR polydioxanone OR poliglecaprone OR PDS or monocryl or PGA) (caesarean OR cesarean OR laparotom*) 1 AND 2 AND 3 Web of Science: (bovine OR cow OR cows OR bovines OR bos) (vicryl OR polyglactin OR catgut OR polyglycolic OR suture material OR polydioxanone OR poliglecaprone OR PGA OR PDS OR monocryl) (caesarean OR cesarean OR laparotomy OR laparotomies) 1 AND 2 AND 3
Dates searches performed:	3 April 2025

**Exclusion / Inclusion Criteria:** 

Exclusion:	Papers not available in English. Expert opinions. Papers studying species other than bovines. Full texts not accessible online or through library services. Papers not related to the PICO question.
Inclusion:	Peer-reviewed studies. Controlled trials.

**Search Outcome:** 

Database	Number of results	Excluded – not relevant to PICO	Excluded – non-English language	Excluded – expert opinion	Excluded – full text not available	Total relevant papers
CAB Abstracts	24	16	3	1	1	3
PubMed	17	15	1	0	0	1
Web of Science	19	10	4	2	0	3
Total relevant papers when duplicates removed						3

## ORCiD

Tom Woods: 0000 0002 3192 7525

## Conflict of Interest

The authors declare no conflicts of interest.

## Disclaimer

Knowledge Summaries are a peer-reviewed article type which aims to answer a clinical question based on the best available current evidence. It does not override the responsibility of the practitioner. Informed decisions should be made by considering such factors as individual clinical expertise and judgement along with patient’s circumstances and owners’ values. Knowledge Summaries are a resource to help inform and any opinions expressed within the Knowledge Summaries are the author's own and do not necessarily reflect the view of the RCVS Knowledge. Authors are responsible for the accuracy of the content. While the Editor and Publisher believe that all content herein are in accord with current recommendations and practice at the time of publication, they accept no legal responsibility for any errors or omissions, and make no warranty, express or implied, with respect to material contained within. For further information please refer to our Terms of Use.

## References

[BIBR-1] Bakkum E.A., Dalmeijer R.A.J., Verdel M.J.C., Hermans J., Blitterswijk C.A., Trimbos J.B. (1995). Quantitative analysis of the inflammatory reaction surrounding sutures commonly used in operative procedures and the relation to postsurgical adhesion formation. Biomaterials.

[BIBR-2] Biondo-Simões M.D.L.P., Oda M.H., Pasqual S., Robes R.R. (2018). Comparative study of polyglactin 910 and simple catgut in the formation of intraperitoneal adhesions. Acta Cirúrgica Brasileira.

[BIBR-3] Bohy A. (2001). Caesarean section in cows: should we regret the loss of catgut?. Le Point Vétérinaire.

[BIBR-4] Boucoumont D., Lecuyer B., Rosentiehl D., Tisserand R., Troccon B., Oullier R. (1979). Effect of caesarean section on fertility of cows. Comparative study of catgut and a polyglycolic acid thread. Le Point Vétérinaire.

[BIBR-5] Dhindsa S.S., Dhaliwal G.S., Ghuman S.P.S., Sood N.K. (2010). Alterations in uterine and peritoneal fluid cytology as well as uterine histopathology following caesarean operation with reference to suture material and intra-peritoneal lubricant in bovines. The Indian Journal of Animal Sciences.

[BIBR-6] Dhindsa S.S., Dhaliwal G.P.S., Ghuman S.P.S. (2010). Prevention of uterine adhesion formation following caesarean section in bovines. The Indian Journal of Animal Sciences.

[BIBR-7] Gomes M.E., Reis R.L. (2004). Biodegradable polymers and composites in biomedical applications: from catgut to tissue engineering. Part 1 Available systems and their properties. International Materials Reviews.

[BIBR-8] Kumar D., Satish, Purohit G.N. (2022). Cesarean section in cattle: A review. Agricultural Reviews.

[BIBR-9] Langley-Hobbs S.J. (2014). Sutures and general surgical implants. Elsevier.

[BIBR-10] Lyons N.A., Karvountzis S., Knight-Jones T.J.D. (2013). Aspects of bovine caesarean section associated with calf mortality, dam survival and subsequent fertility. The Veterinary Journal.

[BIBR-11] Mijten P., Kruif A., Weyden G.C., Deluyker H. (1997). Comparison of catgut and polyglactin 910 for uterine sutures during bovine caesarean sections. Veterinary Record.

[BIBR-12] Newman K.D., Anderson D.E. (2005). Cesarean section in cows. Veterinary Clinics of North America: Food Animal Practice.

[BIBR-13] Singh S., Maxwell D. (2006). Tools of the trade. Best Practice & Research Clinical Obstetrics & Gynaecology.

[BIBR-14] Zaag E.V.D., Klein W.R. (1995). Suture materials and sutures for laparotomy incisions in horses and cattle. Tijdschrift voor Diergeneeskunde.

